# Sleep Deprivation is associated with autophagic impairment and Alzheimer's‐related pathology in the orexinergic ‐ locus coeruleus ‐ hippocampal circuit

**DOI:** 10.1002/alz70855_103832

**Published:** 2025-12-24

**Authors:** Darcy Wear, Christopher D Morrone, Haung (Ho) Yu

**Affiliations:** ^1^ University of Toronto, Toronto, ON, Canada; ^2^ CAMH, Toronto, ON, Canada; ^3^ University of Toronto, Pharmacology and Toxicology, Toronto, ON, Canada

## Abstract

**Background:**

Sleep impairments has been proposed to contribute to Alzheimer's disease (AD) progression, though understanding of the mechanism and association with neural networks remains relatively unexplored. This presentation identifies the relationship between chronic and acute sleep disruption, and relationship to accelerated AD pathology. We propose that autophagic impairment is prominent loss of sleep and circadian rhythmicity, and examine the impact on proteostasis, cognition and AD pathologies.

**Methods:**

We chronically disrupted sleep in 6‐month APPNL‐G‐F and acutely in 4/8/12‐month in APPNL‐G‐FxMAPT (DKI) mice before assessing behaviour, stress and pathological hallmarks of autophagy and AD to identify changes associated with sleep impairment.

**Results:**

Sleep disruption, both acute and chronic, impaired sleep patterns and resulted in behavioral deficits. In acute sleep impairment modeling in DKI mice, we noted hippocampal‐based AD and autophagy changes, alongside increased p62 aggregates in hypothalamic pathology along with neuronal loss. Orexinergic (hypothalamus), but not GABAergic neurons (GAD67, preoptic area), were vulnerable to autophagic disruption. With aging, we observed neuronal loss and preoptic excitatory neuronal p62 increase. In early‐stage dKI, neurons in the locus coeruleus had slight increases in p62+/PHF1+ tau deposition, which were robust at late‐stage. In hippocampal and cortical memory circuits, p62 deposition is predominantly axonal/dendritic, greater in female mice and preceded hippocampal neurodegeneration at later stages, with tau pathology greater in male dKIs. Circadian arrhythmicity and loss of rapid eye movement sleep begins in early‐stage dKI mice and declines over age, more prominently in females.

Under chronic sleep loss, we noted elevated corticosterone in female mice that increased neurodegeneration as seen by reduced mature neurons and increased p62 in the hippocampus, indicative of impaired proteostasis. Males, however, demonstrated autophagic impairments through reductions in the autophagy‐initiating ATG5‐12 complex as well as increased levels of hippocampal phospho‐tau, amyloid plaque deposition and probable neuroinflammatory response.

**Conclusion:**

We identify that both acute and chronic sleep disruption can impair autophagic function in the orexinergic and LC sleep‐regulating regions, negatively impacting behavior. We further note linkages to stress and sex‐based differences that are relevant to the etiology of AD and may represent the need for targeted approaches to treatment in the future.

